# Editorial Note: How sex chromosomes get trapped into nonrecombination

**DOI:** 10.1371/journal.pbio.3003107

**Published:** 2025-03-27

**Authors:** The PLOS Biology Editors

This Primer [1] discusses a model presented in a Research Article by Jay et al. [2] published in the same issue of *PLOS Biology*.

This Editorial Note informs readers that the Research Article [2] (discussed in this Primer as reference 4) has been retracted [3].

The author notes that the processes initially reported in the retracted article and discussed in this Primer have individually also been reported in other studies, notably the role of inversions in establishing recombination suppression [4] and the sheltering of deleterious mutations [5], and continue to be debated.
