# Correction: Editorial Note: How sex chromosomes get trapped into nonrecombination

**DOI:** 10.1371/journal.pbio.3003943

**Published:** 2026-08-11

**Authors:** The PLOS Biology Staff

## Notice of Republication

This Editorial Note [1] was republished on July 27, 2026, to correct errors in the XML that impacted linking to the related article [2].

The publisher apologizes for the errors.
